# Teneurin C-Terminal Associated Peptide (TCAP)-1 and Latrophilin Interaction in HEK293 Cells: Evidence for Modulation of Intercellular Adhesion

**DOI:** 10.3389/fendo.2019.00022

**Published:** 2019-02-01

**Authors:** Mia Husić, Dalia Barsyte-Lovejoy, David A. Lovejoy

**Affiliations:** ^1^Department of Cell and Systems Biology, University of Toronto, Toronto, ON, Canada; ^2^Structural Genomics Consortium, University of Toronto, Toronto, ON, Canada

**Keywords:** TCAP, teneurin, latrophilin, LPHN, GPCR, receptor-ligand interaction, peptides, adhesion

## Abstract

The teneurins are a family of four transmembrane proteins essential to intercellular adhesion processes, and are required for the development and maintenance of tissues. The Adhesion G protein-coupled receptor (GPCR) subclass latrophilins (ADGRL), or simply the latrophilins (LPHN), are putative receptors of the teneurins and act, in part, to mediate intercellular adhesion via binding with the teneurin extracellular region. At the distal tip of the extracellular region of each teneurin lies a peptide sequence termed the teneurin C-terminal associated peptide (TCAP). TCAP-1, associated with teneurin-1, is itself bioactive, suggesting that TCAP is a critical functional region of teneurin. However, the role of TCAP-1 has not been established with respect to its ability to interact with LPHN to induce downstream effects. To establish that TCAP-1 binds to LPHN1, a FLAG-tagged hormone binding domain (HBD) of LPHN1 and a GFP-tagged TCAP-1 peptide were co-expressed in HEK293 cells. Both immunoreactive epitopes were co-localized as a single band after immunoprecipitation, indicating an association between the two proteins. Moreover, fluorescent co-labeling occurred at the plasma membrane of LPHN1 over-expressing cells when treated with a FITC-tagged TCAP-1 variant. Expression of LPHN1 and treatment with TCAP-1 modulated the actin-based cytoskeleton in these cells in a manner consistent with previously reported actions of TCAP-1 and affected the overall morphology and aggregation of the cells. This study indicates that TCAP-1 may associate directly with LPHN1 and could play a role in the modulation of cytoskeletal organization and intercellular adhesion and aggregation via this interaction.

## Introduction

The teneurins are a family of type II transmembrane proteins critical for the development and maintenance of the central nervous system in both vertebrates and invertebrates. Vertebrates contain four paralogous teneurins (teneurin-1 through -4), each of which are 2,500–2,800 residues in length and are comprised of numerous multifunctional domains involved in adhesion, cytoskeletal binding, and other protein-protein interactions (1–5). In both vertebrates and invertebrates, the teneurins have been implicated in the formation of filopodia and outgrowth of neurites, as well as neuronal mapping, axonal path-finding, and increased cell-cell adhesion (6–15).

At the distal end of its extracellular carboxy terminus, each of the teneurins contains a conserved peptide sequence named the teneurin C-terminal associated peptide (TCAP) (16, 17). The four vertebrate TCAP paralogues have notable primary structure similarity to that of corticotropin-releasing factor (CRF), calcitonin and most other Secretin G protein-coupled receptor (GPCR) ligands (16–18). TCAP-1, the most studied of the vertebrate TCAP paralogues to date, is known to be expressed as an independent mRNA that yields a 15 kDa pro-TCAP-1 peptide which may then be processed into the mature 4.7 kDa TCAP-1 (19). The mature TCAP-1 peptide has a number of biological actions independent from teneurin-1. TCAP-1-treated murine immortalized hippocampal and hypothalamic cells show a marked increase in neurite and filopodia production, which is associated with an increased expression of the cytoskeletal components β-actin and β-tubulin. TCAP-1 treatment also increases neurite sprouting, axon fasciculation, and modifies dendrite arborization (19, 20). Similar observations have been made *in vivo*, with CA1 hippocampal neurons exhibiting greater dendritic spine density upon treatment with TCAP-1 (21). TCAP-1 regulates these cytoskeletal changes through activation of the MEK/ERK-1/2 signaling pathway, ultimately leading to modulation of microtubule formation and actin polymerization (22). Additionally, TCAP-1 administration in various rodent models significantly alters anxiety- and stress-related behaviors in acoustic startle response, elevated plus maze, and cocaine-reinstatement studies, further cementing its neuromodulatory roles (21, 23–26). Yet despite the high efficacy TCAP-1 shows both *in vitro* and *in vivo*, the precise mechanism by which these actions occur is not well-understood.

Recent studies indicate that the teneurins are endogenous ligands of Adhesion GPCR subfamily L/latrophilin (ADGRL), or simply, latrophilin (LPHN) (8, 27, 28). The LPHNs are a family of three Adhesion GPCRs found in both vertebrates and invertebrates, and, until the discovery of their interaction with teneurin, were considered orphan receptors, as their only prior known ligand was the exogenous α-latrotoxin, the toxic component of black widow spider venom (29). The binding between teneurin and LPHN1 involves the teneurin C-terminal region and at least the lectin-like domain, olfactomedin-like domain, and the serine-threonine rich region of the LPHN1 extracellular tail, which come together to form a trans-synaptic complex that mediates neuronal cell adhesion and signaling (8, 27, 28, 30). In rat hippocampal cell isolates, LPHN1 and teneurin-2 co-occur at synapses, with LPHN1 primarily being located on the presynaptic membrane, whereas teneurin-2 is primarily found post-synaptically (8). LPHN1-expressing Nb2a neuroblastoma cells preferentially aggregate with those expressing teneurin-2, with the proteins co-localizing at points of cell contact to interact specifically across cell-cell junctions (27). Moreover, co-cultures of HEK293 cells expressing LPHN1 with those expressing either teneurin-2 or teneurin-4 show increased cell aggregate formation, indicating greater adhesion between adjacent cells (8).

Direct interaction between LPHN1 and TCAP-1 has not yet been ascertained; however, both structural and functional evidence suggests that this interaction is likely. Although the extracellular region of teneurin contains a β-barrel formation that partially encapsulates the teneurin C-terminus, the TCAP sequence-containing tip of this region emerges from the barrel and is exposed to the extracellular environment (30), placing TCAP in a favorable position to interact with LPHN1. Furthermore, the three vertebrate LPHN paralogues each contain an extracellular hormone binding domain (HBD) with high sequence similarity to the peptide-binding region of many Secretin GPCRs, such as CRF receptors 1 (CRFR1) and 2 (CRFR2), and are thought to be involved in LPHN ligand binding (31, 32). As the four TCAP paralogues contain sequence similarity to CRF, it is possible that an interaction between TCAP-1 and LPHN1 may occur through this LPHN domain. Therefore, in this study we examined whether TCAP-1 and LPHN1 can interact directly at the LPHN1 HBD, and if TCAP-1 co-localizes with a labeled variant of LPHN1 in HEK293 cells. The results of these studies suggest that TCAP-1 can interact directly with LPHN1 to modify cell-to-cell adhesion and cytoskeletal organization.

## Materials and Methods

### Cell Culture

HEK293 cells were grown on 10-cm culture plates in 12 ml McCoy's 5A medium containing L-glutamine (Gibco) supplemented with 10% heat-inactivated fetal bovine serum (FBS; Invitrogen), 100 U/ml penicillin and 100 μg/mL streptomycin. Cells were maintained at 70–90% confluency at 37°C in a humidified CO_2_ incubator during growth. To passage, the cells were rinsed with phosphate buffered saline (PBS) prior to treatment with 3 mL trypsin for 1–2 min. Four mL of fresh medium was then added to inactivate the trypsin and the cells were centrifuged at 16,000 rpm for 3 min. The supernatant was aspirated, and the cell pellet re-suspended in 5 mL of fresh medium. Cells were re-seeded at 100,000 cells per 10 cm plate in 12 mL of fresh culture medium and allowed to grow for 2–3 days. Prior to all experimentation, cells were grown to 50–80% confluency in 6-well culture plates and serum-starved for 3 h in 2 mL of culture medium without FBS but with penicillin and streptomycin.

### Sequence Alignments

The HBD amino acid sequences of the murine LPHN1-3 (acc#: NP_851382.2, NP_001074767.1, NP_941991.1, respectively) and Secretin GPCRs CRFR1 (acc#: NP_031788.1), CRFR2 (acc#: NP001275547.1), calcitonin receptor (CALCR; acc#: NP_031614.2) and calcitonin gene-related peptide receptor (CGRPR; acc#: NP_061252.2) were obtained from the National Center for Biotechnology Information protein database. A multiple sequence alignment of the HBDs was then performed using the multiple sequence comparison by log-expectation (MUSCLE) alignment tool ver. 3.8 (33).

### Constructs and Transfection

**Figure 1:**
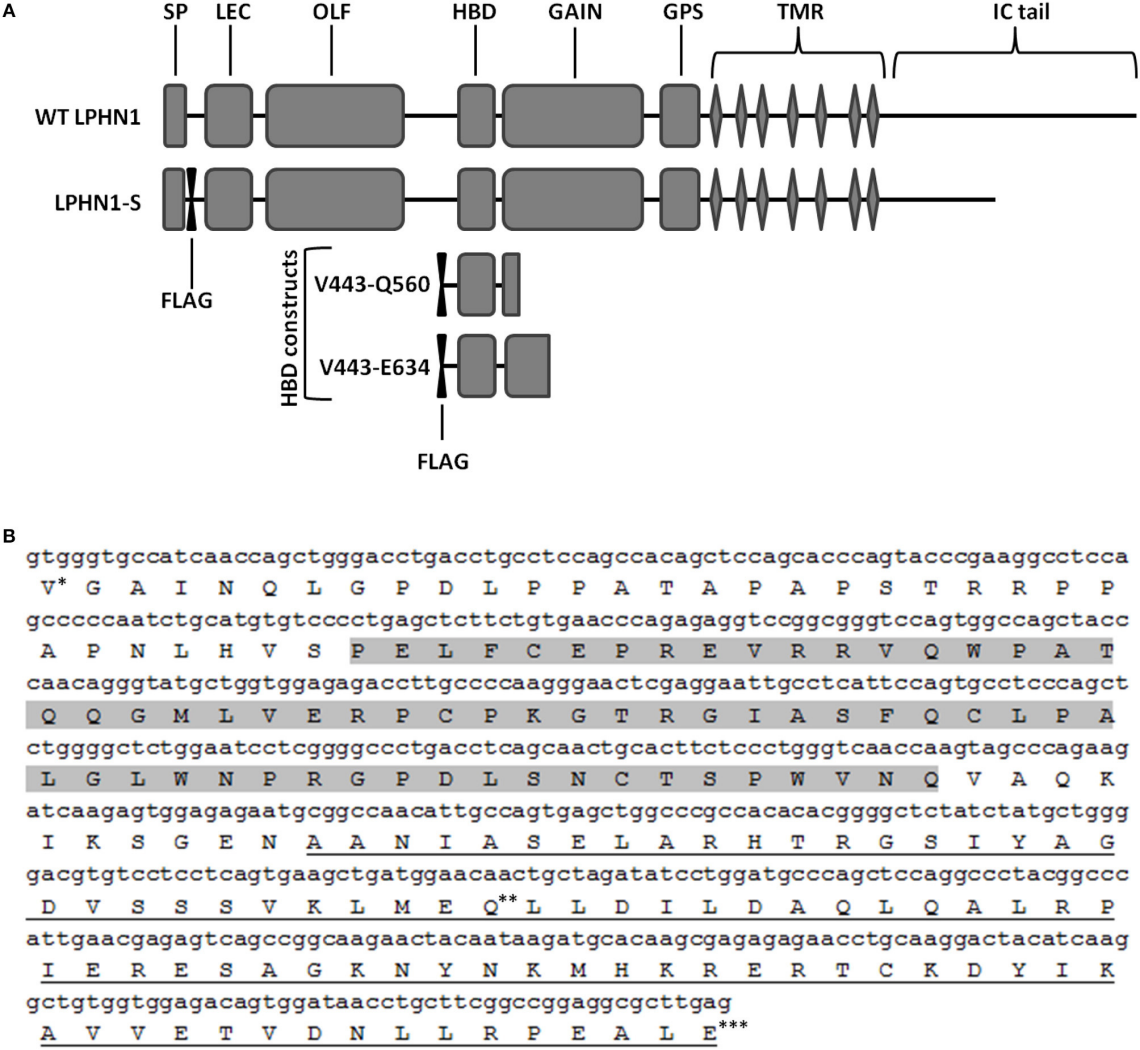
Schematic of the wild-type LPHN1 and the constructs expressed in HEK293 cells. **(A)** Wild-type (WT) LPHN1 contains, on its extracellular region, a signal peptide (SP), a lectin domain (LEC), an olfactomedin-like domain (OLF), a hormone binding domain (HBD) and a GPCR autoproteolysis inducing (GAIN) domain, a GPCR proteolytic site (GPS), followed by a 7-transmembrane region (TMR) and an intracellular (IC) tail. The LPHN1 construct expressed in HEK293 cells, LPHN1-S, contains a FLAG tag downstream of the SP and a truncated intracellular (IC) tail. Constructs used for the co-immunoprecipitation assay of the LPHN1 HBD and TCAP-1 and their constituent domains are also indicated (HBD constructs). Each construct contains an N-terminal FLAG tag. The range of amino acid residues surrounding the HBD domain in each construct is indicated in the construct names. **(B)** Sequence of the LPHN1 region used for co-immunoprecipitation of TCAP-1. Construct V444-Q579 is composed of Valine 444 (^*^) to Glutamine 579 (^**^); construct V444-E634 is composed of residues Valine 444 (^*^) to Glutamic acid 634 (^***^). Gray highlight: HBD; Underline: GAIN domain.

To assess the interaction between LPHN1 and TCAP-1, a LPHN1 construct based on a splice variant of murine LPHN1 (acc#: XM_006531122.2) was expressed in HEK293 cells via lentiviral transfection (Figure 1A). LPHN1 cDNA from the Mammalian Gene Collection clone BC085138 was PCR-amplified using the forward primer GATCACCGGTGCCACCATGGCCCGCTTGGCTGCA and the reverse primer GATCGTCGACTCAGGAGTCACCCCAAGGGA containing AgeI and SalI restriction endonuclease sites, respectively. The PCR product was isolated via gel electrophoresis, purified, and digested using AgeI and SalI. This was subsequently sub-cloned into a pRRL vector (original vector from Addgene plasmid 12252, modified to contain a CMV promoter, multiple cloning sites XbaI, BamHI, AgeI, SalI, and an IRES-puromycin cassette). A FLAG-tag sequence was inserted after the LPHN1 signal peptide (SP) sequence (MARLAAALWSLCVTTVLVTSATQGL) using the Q5 Site-Directed Mutagenesis Kit (New England BioLabs). The pRRL SP-FLAG-LPHN1-IRES-puromycin vector was then co-transfected with pMD2.G (Addgene, 12259), pRSV.REV (Addgene, 12253), and pMDLg/pRRE (Addgene, 12251) plasmids into HEK293 cells. Virus particles were harvested after 72 h and used to transfect HEK293 cells (HEK-LPHN1-S). Wild-type HEK293 cells (HEK-WT) were used as a control cell line, while HEK293 cells transfected with a vector containing puromycin only (HEK-Puro) were used as a transfection control. HEK293 cells were then puromycin-selected and the resulting cell populations were verified for LPHN1 expression using immunocytochemistry and western blotting analysis.

To assess the binding of TCAP-1 with the LPHN1 HBD, two constructs encompassing the LPHN1 HBD region with an added N-terminal FLAG tag were designed (Figure 1A, HBD constructs). The constructs spanned LPHN1 residues V444 to either C579 or E634, and, in both cases, included part of the GPCR autoproteolysis-inducing (GAIN) domain of LPHN1 (Figure 1B). The constructs were transiently co-expressed in HEK293 cells along with either a green fluorescence protein (GFP)-tagged mouse pro-TCAP-1 construct (GFP-pro-mTCAP-1) or a GFP-tagged mature TCAP-1 construct (GFP-mTCAP-1), the amino acid sequences of which were determined based on the TCAP-1 mRNA transcript identified by Chand et al. (19). AntiFlag (Sigma) antibody was used for immunoprecipitation. All transient transfections were performed using the X-treme Gene system (Roche).

### LPHN1 HBD-TCAP-1 Immunoprecipitation Assay

Immunoprecipitation assays were performed to assess the interaction of LPHN1 HBD and TCAP-1. Constructs of the LPHN1 HBD region with an added FLAG tag (Figure 1) were designed and transiently expressed in HEK293 cells along with either GFP-mTCAP-1 or GFP-pro-mTCAP-1. The degree of expression of the LPHN1 HBD constructs was confirmed using western blot (data not shown). To determine if TCAP-1 interacts with the HBD, first the HBD construct proteins were isolated using an anti-FLAG antibody. The HBD constructs were then precipitated through a series of wash and centrifugation steps and eluted. The eluate was then resolved via western blot and probed for presence of the GFP tag on TCAP-1 to see if TCAP-1 interacts with either HBD construct.

### Western Blotting

For cell lysate collection, cells were washed once with ice cold PBS and then lysed on ice for 5 min using radio-immunoprecipitation assay buffer (RIPA; Cell Signaling Technology) with added phenylmethylsulfonyl fluoride (PMSF) protease inhibitor. The lysates were then harvested and centrifuged at 14,000 rpm and 4°C for 20 min to remove any debris. The resulting supernatant was collected for further use in western blot analysis.

To determine the protein concentrations of collected cell lysate samples, a Pierce bicinchoninic acid (BCA) protein assay (Thermo Scientific) was performed according to the manufacturer's instructions. Briefly, standards containing known concentrations of bovine serum albumin (BSA) ranging from 0 to 2,000 μg/ml were prepared. 25 μL of the standards and previously collected cell lysates were added to individual wells of a 96 well-plate. 200 μL of working reagent was then added to each well, and the plate was put on a shaker for 30 s to allow the samples and reagent to sufficiently mix. The plate was then incubated at 37°C for 30 min. Absorbance levels of the standards and samples were measured at 562 nm using a Spectramax Plus Microplate Reader (Molecular Devices). Absorbance values of the standards were then used to create a standard curve from which protein concentrations of cell lysates could be interpolated. Once lysate protein concentrations were determined, all lysates were normalized to provide an equal protein concentration across all samples prior to proceeding with Western blot analysis. Lysates were stored at −20°C.

The expression of LPHN1 in HEK293 cells was determined by western blot. 15 μL of sample were combined with Tricine sample loading buffer (Bio-Rad) containing 2% β-mercaptoethanol. All samples were resolved via 12% sodium dodecyl sulfate polyacrylamide gel electrophoresis (SDS-PAGE) at 100 V for 1 h. The peptides were then electro-transferred onto a Hybond ECL nitrocellulose blotting membranes (Amersham) at 100 V for 75 min. The membranes were washed 3x for 10 min with PBS and blocked in a 5% BSA-PSBT solution (5% BSA w/v dissolved in PBS with 0.2% Tween®20) on a shaker for 1 h at RT. They were then incubated in 5% BSA-PBST with goat polyclonal LPHN1 primary antibody (Santa Cruz) at a 1:1,000 dilution overnight at 4°C with gentle agitation. Immunoprecipitation immunoblotting and GFP detection was performed using mouse anti-GFP (1:5,000, Clontech). The following day, the membranes were given 3x 10 min washes with PBST and incubated for 1 h at RT in 1% milk PBST (1% dehydrated milk w/v dissolved in PBST) containing horseradish peroxidase-linked donkey anti-goat secondary antibody (Santa Cruz) at a 1:7,500 dilution or IR800 conjugated anti-mouse secondary antibody (LiCor). The membranes were then washed 3x for 10 min with PBST prior to a 1 min incubation in chemiluminescence reagent (ECL, Amersham). For protein detection, the membranes were exposed onto ECL Hyperfilm (VWR) for 0.5–6 min. IR800 signal was visualized on Odyssey scanner (LiCor). Western blot gel images were quantified using Fiji software (34), and statistical analysis was performed using GraphPad Prism 7.

### Peptide Synthesis

Mouse TCAP-1 (mTCAP-1) was synthesized at 95% purity using f-moc-based solid phase synthesis. mTCAP-1 with an arginine (R) to lysine (K) substitution at position 37 (K37-mTCAP-1) was synthesized as previously described (17). K37-mTCAP-1 was further tagged with Fluorescein (FITC) (Thermo Scientific) using *N*-hydroxysuccinimide according to the manufacturer's instructions. Briefly, the K37-mTCAP-1 was solubilized in borate buffer while the Fluorescein dye was dissolved in dymethylformamide (DMF). A 20-fold molar excess of the dye was added to the peptide and the solution was incubated at RT for 1 h in the dark. The solution was then passed through polyacrylamide desalting columns (Thermo Scientific) for purification and 8 fractions were collected. Protein absorbance of the fractions was measured at 280 and 495 nm using a Spectramax Plus Microplate Reader (Molecular Devices) to determine which fractions contained the highest protein content. The fractions with the highest absorbance readings, indicating the highest FITC-tagged K37-mTCAP-1 (FITC-K37-mTCAP-1) content, were then combined and stored as aliquots at −20°C.

### Immunocytochemistry

For all immunocytochemical (ICC) analysis, HEK-WT, and HEK-LPHN1-S cells were first grown on poly-D-lysine coated cover slips to 50–80% confluency. To confirm successful LPHN1 transfection, cells were first fixed onto the cover slips via treatment with 4% paraformaldehyde (PFA) for 20 min. They were then washed 3x with PBS, permeabilized with 0.3% Triton X-100 (Sigma Aldrich) and washed again 3x with PBS prior to blocking with PBS containing 10% v/v normal goat serum (NGS) for 1 h. The cells were incubated for 1 h with Cy3-tagged Flag antibody (Sigma Aldrich), given 3x PBS washes and mounted onto microscope slides using VECTASHIELD Mounting Medium with DAPI (Vector Laboratories).

LPHN1 and FITC-K37-mTCAP-1 co-localization studies were done by first incubating HEK-WT and HEK-LPHN1-S cells in culture medium containing 20 nM HEPES buffer (pH 7.4), 0.1% BSA and FITC-K37-mTCAP-1 at a 1:400 dilution for 12 h at 4°C. The cells were then given 3 washes with cold culture medium, fixed with 4% PFA and washed 3x with ice cold PBS. Subsequently, the cells were blocked for 1 h at RT with PBS containing 3% w/v BSA and incubated with Cy3-tagged FLAG antibody (Sigma Aldrich) for 1 h. Following this, they were washed 3x with 3% BSA blocking solution and once with water, and then finally mounted onto microscope slides as described above.

For morphology studies involving cell membrane staining with wheat germ agglutinin (WGA), cells were first fixed for 10 min using 4% PFA, and then washed 3x with Hank's Balanced Salt Solution (HBSS; Gibco) prior to incubation with HBSS containing WGA (Invitrogen) at a 1:1,000 dilution for 10 min. The cells were washed 2x with HBSS, permeabilized using 0.2% Triton X-100 for 10 min, washed 3x with PBS and mounted onto microscope slides as described above.

To examine cytoskeletal morphology, cells were either first treated with 100 nM TCAP-1 or vehicle for 1 h or immediately washed 3x with PBS and incubated in a solution containing 1 mL of 4% PFA, 10 μL Triton X-100, and 15 μL of the filamentous actin (f-actin) probe Alexa Fluor 594 Phalloidin (Life Technologies) for 10 min. The cells were then washed 3x with PBS and mounted onto microscope slides as described above.

All cell imaging was done using confocal microscopy (TCS SP8, Leica Microsystems) with 40x, 63x, or 100x oil immersion objectives. Image acquisition settings were calibrated to control cell groups (non-TCAP-1-treated HEK-WT cells). After acquisition, the images were converted into JPEG format for further analysis. HEK293 nuclear height was measured via Z-stacking from the base of the nucleus to just beyond the top of the nucleus, to acquire a measurement of the full organelle.

### Digital Image Analysis

Immunofluorescence intensity of all digital images of cells was analyzed using Fiji software (34). For whole cell size analysis, each cell that was completely visible within a merged image displaying WGA and DAPI staining as well as the differential interference contrast (DIC) was digitally analyzed. Every cell that was clearly visible in an ICC image was individually isolated using the Fiji freehand selection tool, and the area and perimeter of the cells were obtained and averaged. To analyze nuclear size, DAPI nuclear stain images were used. First, a color threshold was set to create multiple regions of interest (ROIs) based on blue pixel intensity. This allowed for simultaneous isolation of multiple nuclei within a single image. Any single ROIs consisting of more than one nucleus or of cells in the process of mitosis (as indicated by anaphase-like chromosomal arrangement) were discarded and the remaining ROIs were measured for their perimeter and area. Nuclei that were not captured by this method were individually isolated using the Fiji freehand selection tool and their area and perimeter were measured using the same method as for whole cell measurements.

FITC-K37-mTCAP-1 uptake by cells was quantified by immunofluorescence intensity, specifically by examining green pixel intensity histograms of confocal microscopy images for HEK-WT, HEK-Puro, and HEK-LPHN1-S cells. Analysis was done by discarding the first 20 intensity values on the histogram, as these corresponded to black pixels, indicating no green FITC tag signal. For each image examined, the total number of cells per image was counted, and the total number of green pixels with an intensity of 20–255 was obtained. This number of pixels was then divided by the number of cells in that picture as a way to account for differences in cell count per image. These values were used for further statistical analysis by GraphPad Prism 7. Cytoskeletal differences between HEK-WT and HEK-LPHN1-S cells were quantified in the same manner using Phalloidin stain images for each cell group and red pixel intensity histograms.

### Statistical Analysis

All results are represented as a mean ± standard error of the mean (SEM). An *a priori* hypothesis of *p* < 0.05 was utilized for all analyses. The data was analyzed with GraphPad Prism 7 using either a two-tailed *t*-test or one-way or two-way analyses of variance (ANOVA) with a Tukey's *post hoc* test. Mean values were obtained from a minimum of 3 independent repeats of an experiment, where a single repeat refers to cells grown in a single well of a 6-well plate. For digital analysis of ICC images, representative photos of each repeat were analyzed. Cell height measurements were taken from 4 distinct regions of each slide cells were mounted onto, where 4 cells per region were measured for a total of 16 measurements per slide (one repeat). Data was considered statistically significant if *p* < 0.05 (^*^p < 0.05, ^**^p < 0.01, ^***^p < 0.001, ^****^p < 0.0001).

## Results

### Comparison of LPHN and Secretin GPCR HBD Amino Acid Sequences

**Figure 2:**
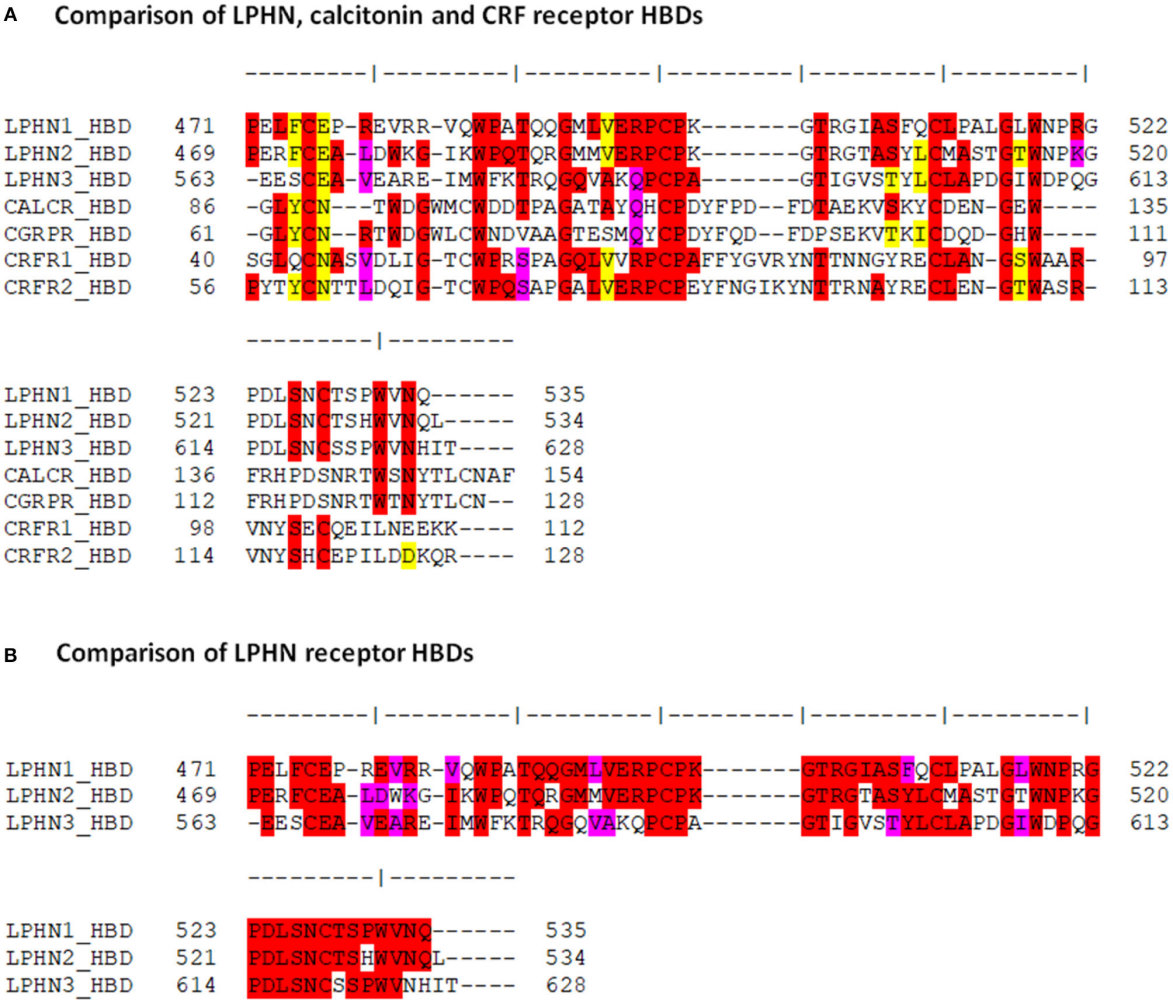
Comparison of the amino acid sequences among the LPHN, calcitonin and CRF HBDs. **(A)** Amino acid sequence alignment of the HBDs for murine LPHN, calcitonin, and CRF receptors. **(B)** Alignment of the putative HBDs for the three LPHN receptors. Residue identity is indicated in red, conservative substitutions are indicated in pink, and homologous replacements are indicated in yellow.

The putative HBD region of LPHN1 showed about 30% identity at the amino acid level with the HBD regions of the calcitonin and CRF receptors (Figure 2A), confirming the homology of this domain within this receptor group. This was also reflected by conserved residues at LPHN1 positions 475 (C), 485 (W), 492 (G), 499 (C), 500 (P), 511 (C), 516 (G), and 518 (W). With respect to LPHN, the CRF receptors showed a slightly higher degree of identity than the calcitonin receptors, noted by the conservation of residues at LPHN1 positions 598 (P), 526 (S), and 528 (C). Furthermore, at least 50% identity was observed between the 64-residue HBD sequences of the three LPHN paralogues themselves (Figure 2B).

### TCAP-1 Interaction With a LPHN1 HBD Cassette

**Figure 3:**
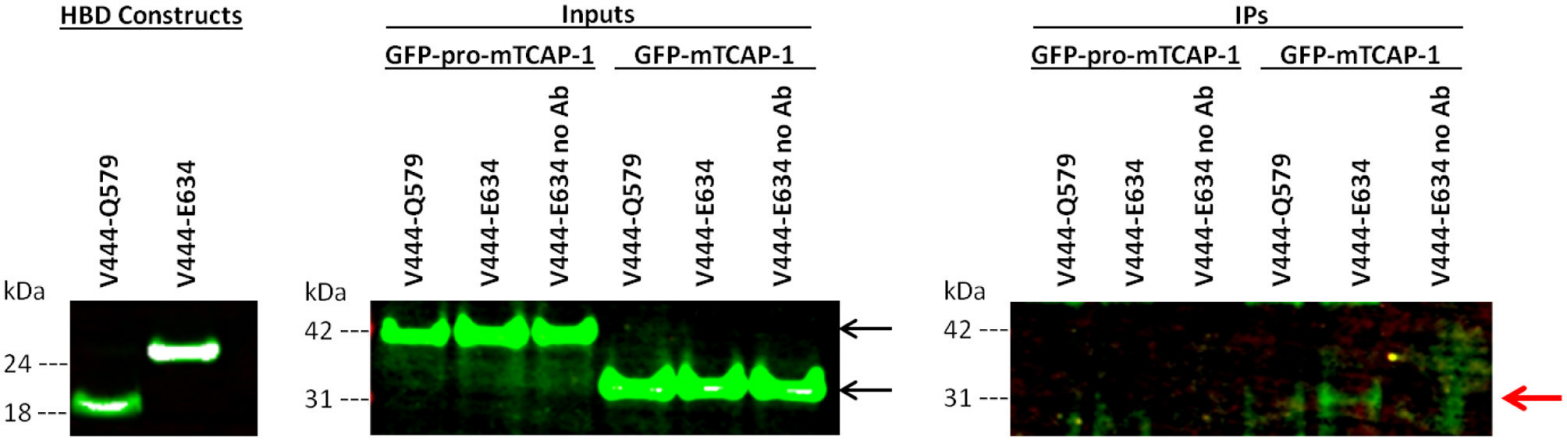
The mature TCAP-1 peptide interacts with the HBD and a partial GAIN domain of LPHN1. **(HBD constructs)** LPHN1 HBD constructs were successfully expressed in HEK293 cells. **(Inputs)** Input lanes indicate strong presence of GFP-pro-mTCAP-1 or mature TCAP-1 in cell lysates prior to immunoprecipitation with HBD constructs V444-Q579 and V444-E634. Expected band size of GFP-pro-mTCAP-1 and GFP-mTCAP-1 are 40 and 30 kDa, respectively (black arrows). **(IPs)** Immunoprecipitation lanes show western blot resolution of corresponding eluates from input lanes. “No Ab” indicates that no anti-FLAG antibody was used to for immunoprecipitation of the HBD construct, serving as a negative control. No bands corresponding to GFP-pro-mTCAP-1 were isolated with either of the HBD constructs used for IP. A faint band at ~30 kDa, corresponding to GFP-mTCAP-1, is present when HBD V444-Q579 was used for IP. A stronger band of the same size is seen when IP was performed using HBD V444-E634 (red arrow). No bands corresponding to the GFP-pro-mTCAP-1 peptide were observed in the IP.

To determine if TCAP-1 interacts directly with the LPHN1 HBD, FLAG-tagged LPHN1 HBD constructs V444-Q579 and V444-E634 (Figure 1) were transiently expressed in HEK293 cells along with GFP-pro-mTCAP-1 and GFP-mTCAP-1 peptides. The HBD constructs were then used as bait proteins in a co-immunoprecipitation (co-IP) assay to determine if either the pro-TCAP-1 or the mature TCAP-1 peptide interacts with the LPHN1 HBD (Figure 3). First, the expression of both GFP-pro-mTCAP-1 and GFP-mTCAP-1 in HEK293 cells were determined (Figure 3, inputs). Western blot bands, at ~40 and 30 kDa, corresponding to the sizes of GFP-pro-mTCAP-1 and GFP-mTCAP-1, respectively, were observed, indicating strong expression of these peptides in their respective cell lines. The results of the co-IP assay (Figure 3, IPs) showed no bands at 40 kDa, corresponding to GFP-pro-mTCAP-1, when either the V444-Q579 or the V444-E634 construct was used as a bait protein. However, bands as 25 and 50 kDa were observed with both constructs (IgG light and heavy chains; data not shown). In contrast to these findings, a band at 30 kDa, corresponding to GFP-mTCAP-1, was observed when the V444-E634 construct was used as bait (Figure 3, IPs). A fainter 30 kDa band could also be seen when the V444-Q579 construct was used. Again, additional bands at 25 and 50 kDa were observed. These results suggest that a stronger affinity of the TCAP-1 construct occurred when a larger proportion of the GAIN domain was included. Control experiments in which no anti-FLAG antibodies were used to precipitate the HBD constructs were also performed where the eluates showed no detectable bands for either GFP-pro-mTCAP-1 or GFP-mTCAP-1 (Figure 3, IPs, “no Ab” lanes).

### Over-Expression of LPHN1 Constructs in HEK293 Cells

**Figure 4:**
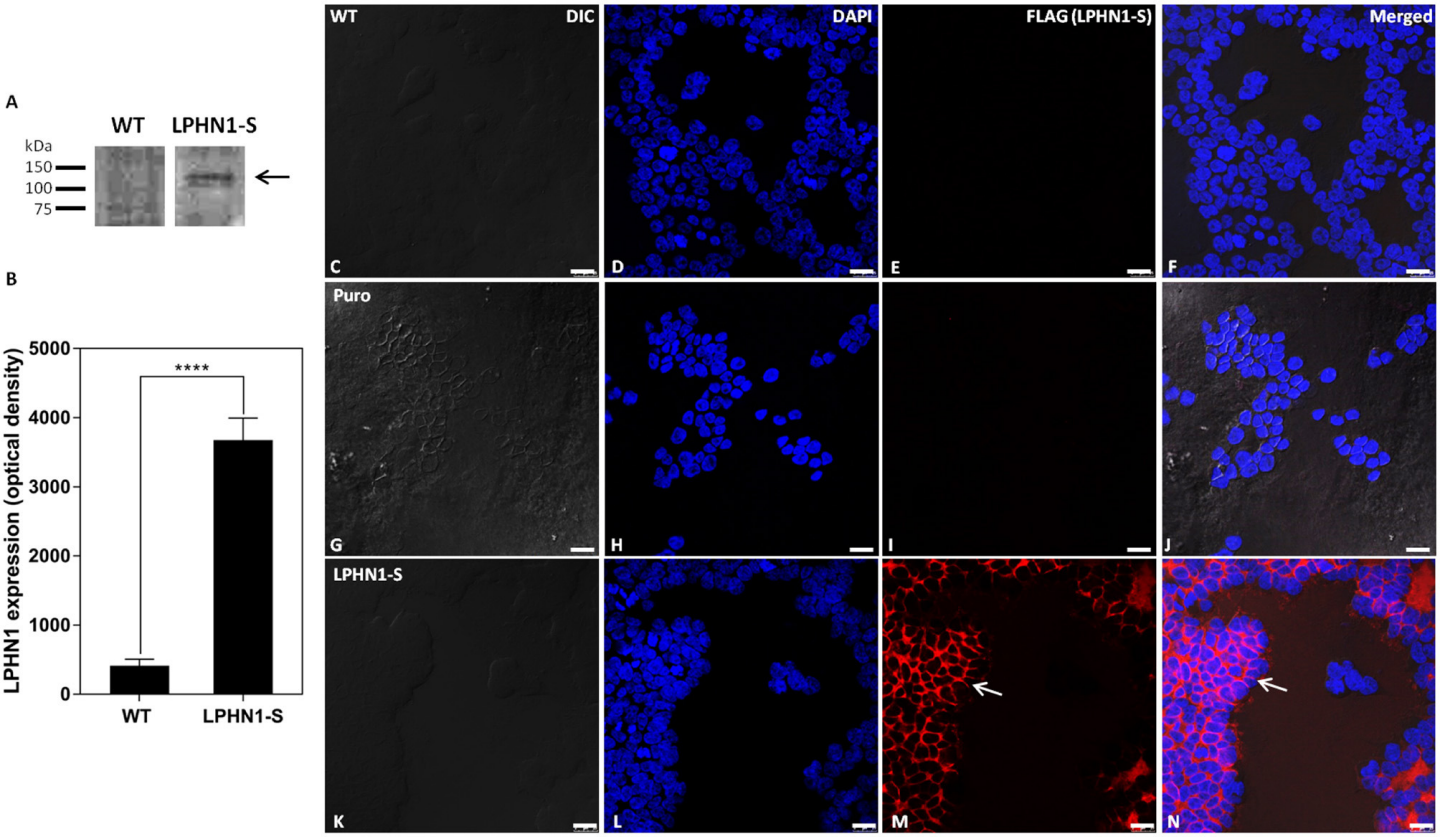
HEK-LPHN1-S cells strongly express LPHN1-S, whereas HEK-WT and HEK-puro cells do not. **(A)** Western blot of LPHN1 expression in HEK-WT and HEK-LPHN1-S cells. A band at ~120 kDa is present in the HEK-LPHN1-S cells, but not in the HEK-WT cells (black arrow). Expected size is 116 kDa. **(B)** Quantification of LPHN1 protein expression in HEK-WT and HEK-LPHN1 cells. (Mean ± SEM; *n* = 4; ^****^p < 0.0001; two-tailed *t*-test) **(C–F)** Confocal images of HEK-WT cells. **(G–J)** Confocal images of HEK-Puro cells. **(K–N)** Confocal images of HEK-LPHN1-S cells. **(C,G,K)** DIC image. **(D,H,L)** DAPI staining of nuclear proteins. **(E,I,M)** FLAG-tagged LPHN1-S. **(F,J,N)** Merged images of **A–C**, **G–I**, and **K–M**, respectively. White arrow in **M,N** indicates FLAG-tagged LPHN1-S expression in HEK-LPHN1-S cells. A strong FLAG signal is seen at the cell membrane of HEK-LPHN1-S cells only, indicating strong expression of the LPHN1-S construct in this region (white arrow). HEK-WT and HEK-Puro cells showed no FLAG signal. Scale bar in all images is 25 μm.

The LPHN1-S construct, containing an N-terminal FLAG tag and a truncated intracellular tail, was expressed in HEK293 cells via lentiviral infection to create a cell line in which the interaction between TCAP-1 and LPHN1 could be examined. Western blot analysis using anti-LPHN1 antibodies was performed to confirm successful over-expression of the LPHN1-S construct in HEK-LPHN1-S cells, and to determine the endogenous degree of LPHN1 expression in HEK-WT cells (Figures 4A,B). A band at 120 kDa was detected in HEK-LPHN1-S cells, but not in HEK-WT cells. It should be noted that endogenous expression of the LPHN2 and LPHN3 isoforms in HEK-WT and HEK-LPHN1-S cells was not examined in this study.

To observe the pattern of LPHN1 expression in these cells, Cy3-tagged anti-FLAG antibodies were used to label the LPHN1-S protein for confocal microscopy imaging (Figure 4). HEK-WT cells showed no detectable FLAG signal (Figure 4E). HEK-Puro vector control cells expressing just a puromycin resistance gene without LPHN1-S also showed no detectable FLAG signal (Figure 4I). The HEK-LPHN1-S cells, however, showed a strong FLAG signal that was localized primarily to the cell membrane (Figures 4M,N, white arrow).

### Binding of TCAP-1 to LPHN1 in HEK293 Cells

**Figure 5:**
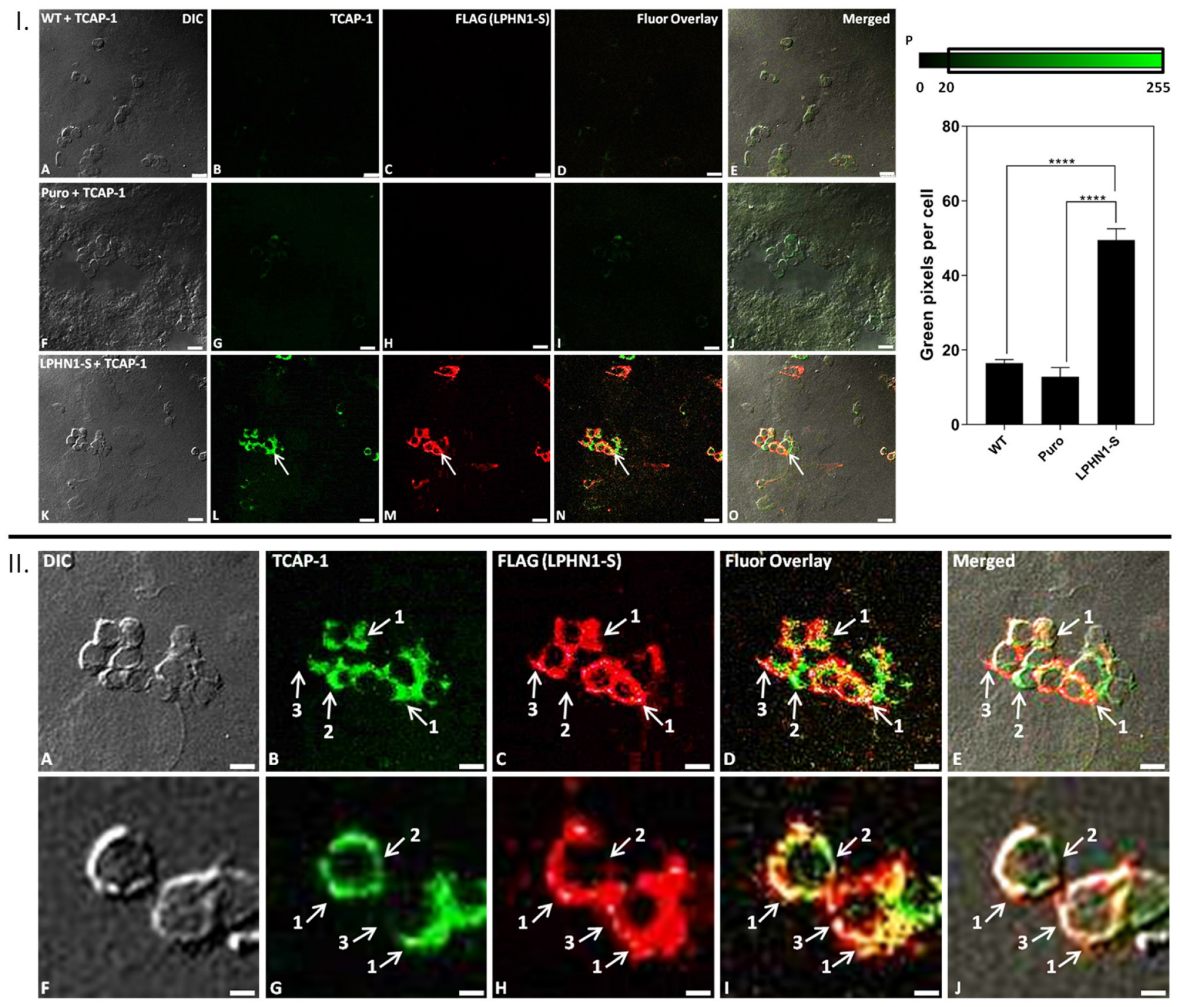
FITC-K37-mTCAP-1 co-localizes with LPHN1 expression. **I: (A–E)** HEK-WT cells treated with FITC-K37-mTCAP-1. **(F–J)** HEK-Puro cells treated with FITC-K37-mTCAP-1. **(K–O)** HEK-LPHN1-S cells treated with FITC-K37-mTCAP-1. Arrows indicate regions of FITC-K37-mTCAP-1 and LPHN1-S co-localization. **(A,F,K)** DIC. **(B,G,L)** FITC-K37-mTCAP-1. **(C,H,M)** FLAG (LPHN1-S) expression. **(D,I,N)** FITC-K37-mTCAP-1 and FLAG (LPHN1-S) fluorescence channel overlay images. **(E,J,O)** Merged image of all channels. A low level of green fluorescence is seen in HEK-WT and HEK-Puro cells, indicating some uptake of FITC-K37-mTCAP-1. However, HEK-LPHN1-S cells show a much brighter green fluorescence signal, indicating a higher uptake of FITC-K37-mTCAP-1. Scale bar in all images is 25 μm. **(P)** Average number of green pixels per cell with an intensity ranging from 20 to 255 in HEK-WT, HEK-Puro, and HEK-LPHN1-S cells treated with FITC-K37-mTCAP-1. Pixel intensity range is indicated above graph. HEK-LPHN1-S cells have a significantly higher number of green pixels with intensities of 20–255 per cell, indicating a higher degree of FITC-K37-mTCAP-1 uptake compared to HEK-WT and HEK-Puro cells. No significant differences between HEK-WT and HEK-Puro cells were observed. (Mean ± SEM; *n* = 5; ^****^p < 0.0001; one-way ANOVA) **II: (A–J)** Confocal images of HEK-LPHN1-S cells. **(A,F)** DIC. **(B,G)** FITC-K37-mTCAP-1. **(C,H)** LPHN1-S. **(D,I)** Merged images of **B–C** and **G–H**, respectively. **(E,J)** Merged images of **A–C** and **F–H**, respectively. Scale bar in **A–E** is 20 μm and in **F–J** is 6 μm. Regions of FITC-K37-mTCAP-1 and LPHN1-S co-localization on the cell membrane were predominant (arrows labeled 1); however, areas of only FITC-K37-mTCAP-1 localization (arrows labeled 2) or of only LPHN1-S localization (arrows labeled 3) were also seen present in HEK293-LPHN1-S cells.

Immunocytochemistry analysis was performed using HEK-WT, HEK-Puro, and HEK-LPHN1-S cells treated with FITC-K37-mTCAP-1 to observe the degree of TCAP-1 uptake in each cell type and to determine if TCAP-1 co-localizes with LPHN1 (Figure 5). FITC-K37-mTCAP-1 and LPHN1 were found exclusively at the cell membrane, with largely overlapping localization patterns (Figures 5I,L–O arrows); however, regions of the cell membrane with just FITC-K37-mTCAP-1 or just LPHN1 fluorescence were also observed (Figure 5II). HEK-WT and HEK-Puro cells had little or low FITC-K37-mTCAP-1 uptake. In contrast, considerable FITC-K37-mTCAP-1 uptake was present in HEK-LPHN1-S cells. The degree of FITC-K37-mTCAP-1 uptake was quantified as a function of green pixels per cell (Figures 5I,P). Treatment with FITC-K37-mTCAP-1 yielded a significant signal increase (green pixels per cell) in HEK-LPHN1-S cells compared to HEK-WT and HEK-Puro cells (WT: 16.491 ± 0.942 pixels/cell; Puro: 12.790 ± 2.536 pixels/cell; LPHN1-S: 49.498 ± 3.042 pixels/cell; *p* < 0.0001).

### Changes in Cell Size Upon LPHN1 Over-Expression

**Figure 6:**
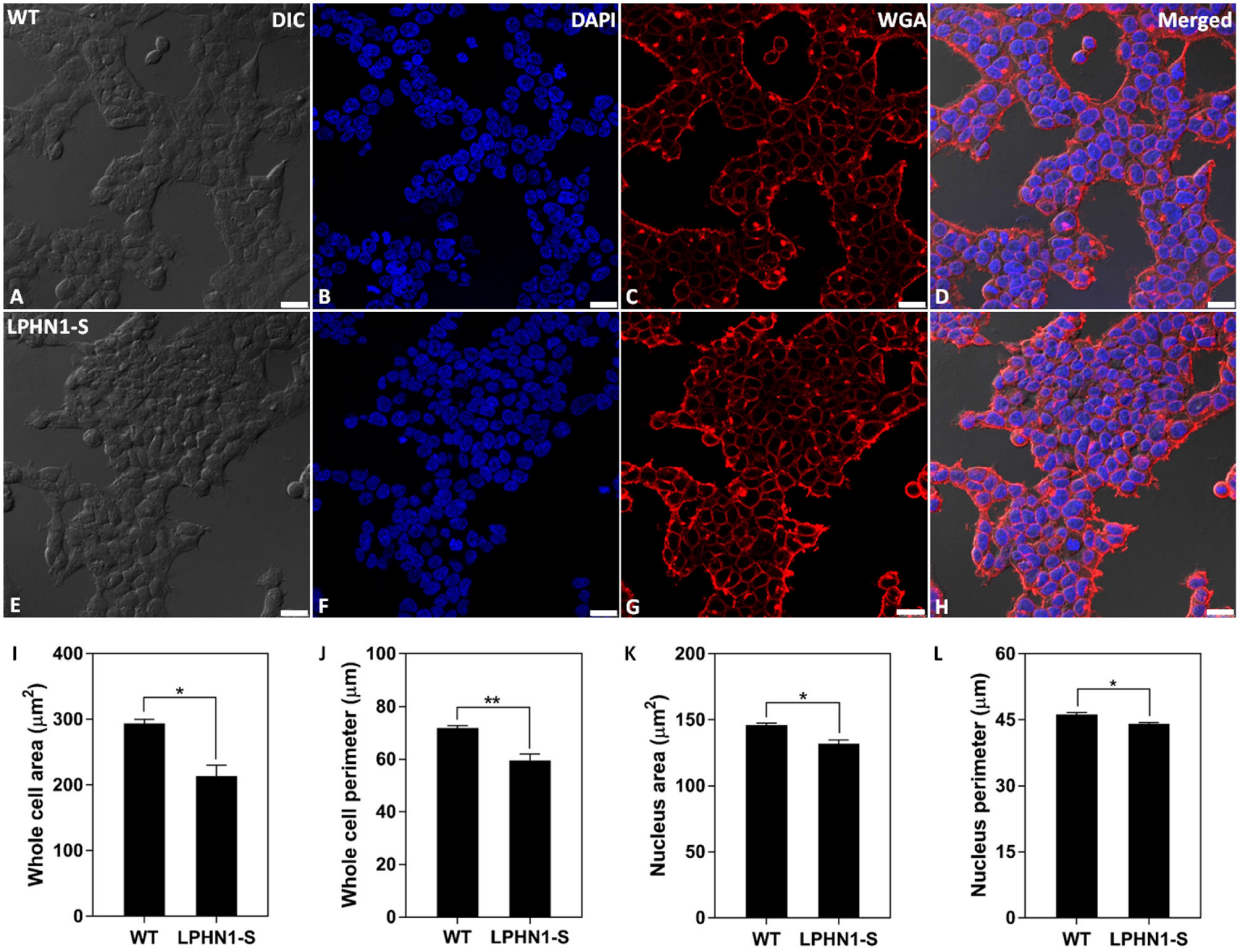
HEK-LPHN1-S cells have a smaller whole cell and nuclear size than HEK-WT cells. **(A–D)** Confocal images of HEK-WT cells. **(E–H)** Confocal images of HEK-LPHN1-S cells. **(A,E)** DIC. **(B,F)** DAPI staining of nuclear protein. **(C,G)** WGA membrane stain. **(D,H)** Merged images of **A–C** and **E–G**, respectively. **(I–L)** Quantification of WT and LPHN1-S HEK whole cell size, whole cell perimeter, nuclear size and nuclear perimeter. HEK-LPHN1-S cells had significantly smaller whole cell area, whole cell perimeter, nuclear area, and nuclear perimeter than HEK-WT cells. Scale bar is 25 μm. (Mean ± SEM, *n* = 3, ^*^p < 0.05, ^**^p < 0.01, two-tailed *t*-test).

To examine the effects of over-expressing LPHN1 on HEK293 cell morphology, HEK-WT and HEK-LPHN1-S cells were labeled with the cell membrane marker WGA and imaged using confocal microscopy (Figures 6A–H). The HEK-LPHN1-S cells appeared to be smaller in diameter and clustered closer together than the HEK-WT cells. However, this observation was due to the initial 2-dimensional nature of this analysis. Subsequently, the morphology of these cells was characterized by quantifying the whole cell and nuclear area and perimeter (Figures 6I–L). HEK-WT cells had an average whole cell area of 293.6 ± 6.2 μm^2^, whereas HEK-LPHN1-S cells had a whole cell area that was 27.2% smaller at 213.7 ± 16.4 μm^2^ (*p* < 0.05; Figure 6I). Similarly, the HEK-LPHN1-S whole cell perimeter of 59.6 ± 2.3 μm was 17% smaller than the HEK-WT cell perimeter of 71.8 ± 0.9 μm (*p* < 0.01; Figure 6J). The HEK-LPHN1-S cell nuclear area of 131.8 ± 3.0 μm^2^ was 9.6% smaller than that of the HEK-WT cells at 146.0 ± 1.4 μm^2^ (*p* < 0.05; Figure 6K). Finally, the HEK-LPHN1-S nuclear perimeter of 44.1 ± 0.3 μm was 4.6% smaller than HEK-WT nuclear perimeter of 46.3 ± 0.4 μm (*p* < 0.05; Figure 6L).

### Changes in Cytoskeletal Organization Upon LPHN1 Over-Expression

**Figure 7:**
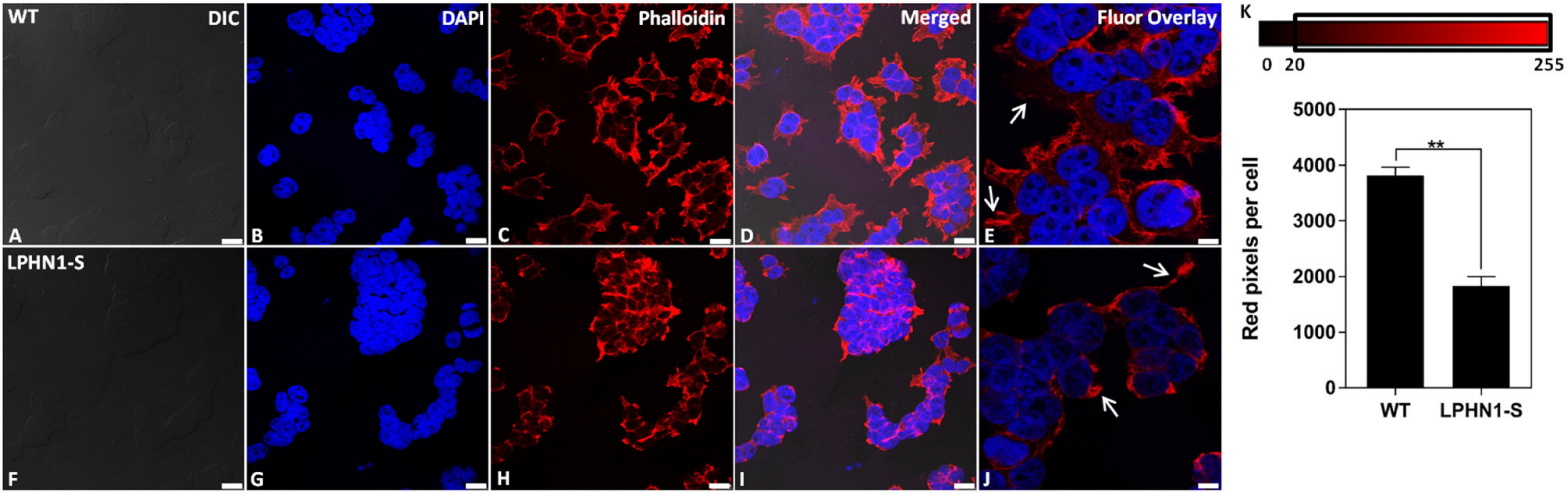
HEK-LPHN1-S cells have decreased f-actin expression compared to HEK-WT cells. **(A–E)** Confocal images of HEK-WT cells. **(F–J)** Confocal images of HEK-LPHN1-S cells. **(A,F)** DIC. **(B,G)** DAPI staining of nuclear proteins. **(C,H)** Phalloidin staining of f-actin. **(D,I)** Merged images of **A–C** and **F–H**, respectively. **(E,J)** DAPI and Phalloidin fluorescence overlay. White arrows indicate f-actin projections. HEK-WT cells are larger than HEK-LPHN1-S cells and express a much greater amount of f-actin. Their f-actin projections appear to be greater in both number and size than those of HEK-LPHN1-S cells. Scale bar in **A–D, F–I** is 25 μm, and in **E,J** is 10 μm. **(K)** Average number of red pixels (f-actin-bound Phalloidin signal) per cell with an intensity of 20–225 (intensity range indicated above). HEK-LPHN1-S cells have significantly fewer red pixels per cell compared to HEK-WT cells, indicating less Phalloidin staining and thus a reduced expression of f-actin. (Mean ± SEM, *n* = 5, ^**^p < 0.01, two-tailed *t*-test).

Next, the f-actin content of HEK-WT and HEK-LPHN1-S cells was assessed as a biomarker to further characterize the morphological differences between these cell types. The f-actin cytoskeleton was fluorescently stained using Phalloidin (Figure 7). HEK-WT cells showed a strong degree of Phalloidin labeling, indicating a large amount of f-actin. HEK-WT cells were much larger than HEK-LPHN1-S cells and had more f-actin projections extending from them compared to HEK-LPHN1-S cells, which had little to no projections of the same morphology (Figures 7E,J, white arrows). Similarly, the HEK-WT cells appeared to be more spread out, with more f-actin between individual cells compared to the HEK-LPHN1-S cells, which had a more clustered appearance with little f-actin between individual cells.

The amount of f-actin present in each cell type was further quantified as a function of red pixels per cell, in which the number of red pixels corresponds to the amount of Phalloidin-bound f-actin present per cell (Figure 7K). HEK-LPHN1-S cells had 52% fewer red pixels per cell than HEK-WT cells (WT: 3818.327 ± 144.874 pixels/cell; LPHN1-S: 1834.192 ± 166.365 pixels/cell; *p* < 0.01), indicating a significantly lower level of f-actin.

### Changes in Cytoskeletal Organization Upon Treatment With TCAP-1

**Figure 8:**
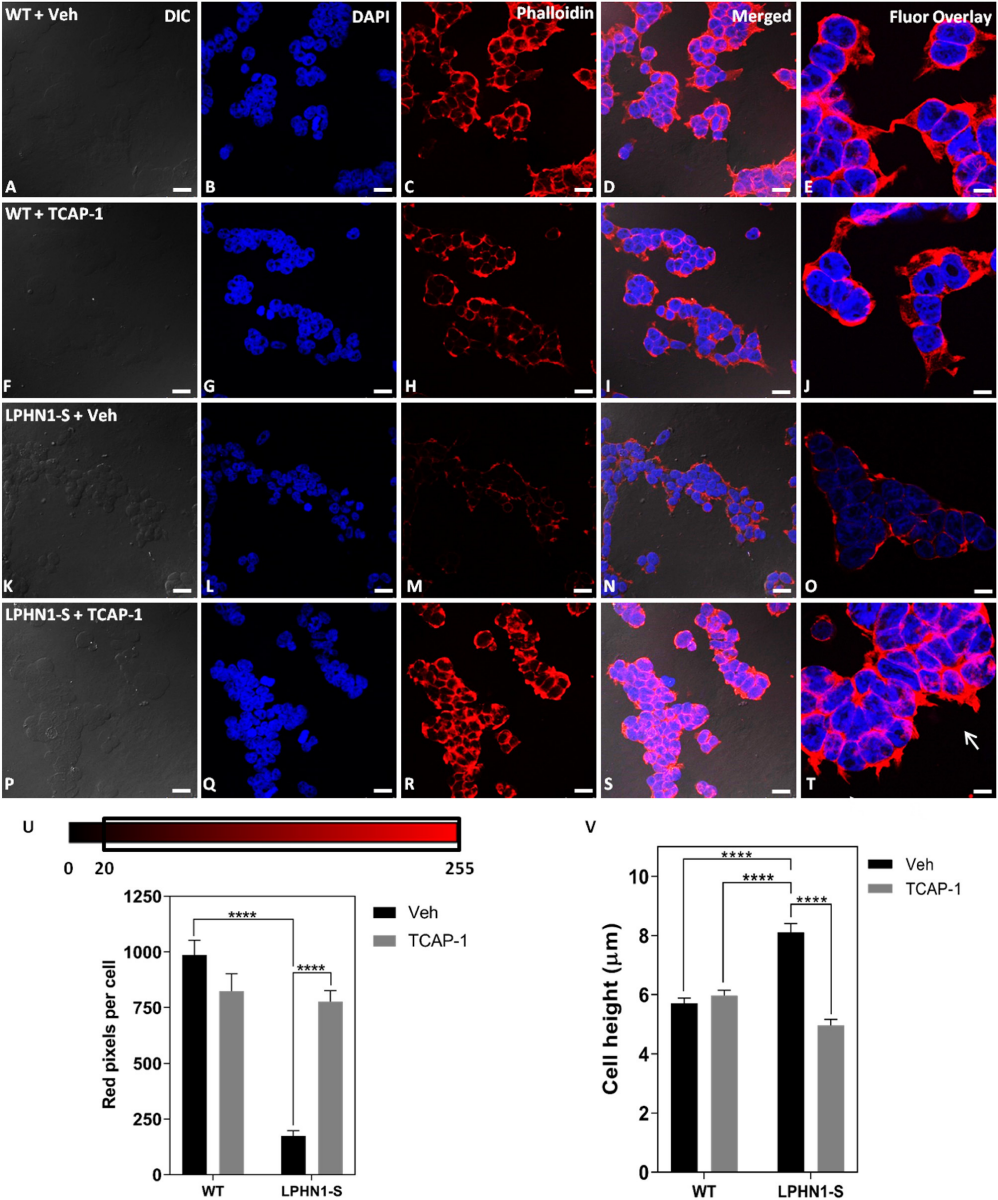
mTCAP-1 treatment induces a strong up-regulation of f-actin and changes in cell height in HEK-LPHN1-S cells but not in HEK-WT cells. **(A–E)** Images of HEK-WT cells treated with vehicle for 60 min. **(F–J)** Images of HEK-LPHN1 cells treated with 100 nM mTCAP-1 for 60 min. **(K–O)** Images of HEK-WT cells treated with vehicle for 60 min. **(P–T)** Images of HEK-LPHN1 cells treated with mTCAP-1 for 60 min. **(A,F,K,P)** DIC. **(B,G,L,Q)** DAPI staining of nuclear proteins. **(C,H,M,R)** Phalloidin staining of f-actin. **(D,I,N,S)** Merged images of **A–C, F–H, K–N**, and **P–S**, respectively. **(E,J,O,T)** DAPI and Phalloidin fluorescence overlay. White arrows in **T** indicate f-actin projections. mTCAP-1 treatment does not cause an increase in f-actin in HEK-WT cells. In HEK-LPHN1-S cells, however, a significant upregulation of f-actin is seen upon treatment with mTCAP-1. Scale bar in **A–D, F–I, K–N, P–S** is 25 μm and in **E,J,O,T** is 10 μm. **(U)** Average number of red pixels (f-actin-bound Phalloidin signal) per cell with an intensity of 20–225 (intensity range indicated above) in HEK-WT and HEK-LPHN1 cells treated with either vehicle (gray) or 100 nM mTCAP-1 (black) for 60 min. Compared to vehicle treatment, mTCAP-1 increased f-actin in HEK-LPHN1-S cells only. (Mean ± SEM; *n* = 5, ^****^p < 0.0001; two-way ANOVA and a Tukey's *post hoc* test) **(V)** Height measurements of HEK-LPHN1-S and HEK-WT cells treated with either vehicle (gray) or 100 nM mTCAP-1 (black) for 60 min. No differences were observed between mean cell heights of vehicle and mTCAP-1 treated HEK-WT cells. Vehicle-treated HEK-LPHN1-S cells were larger than either HEK-WT group. mTCAP-1 treatment of HEK-LPHN1-S cells decreased cell height. (Mean ± SEM; *n* = 3, 4 measurements per n; ^****^p < 0.0001; two-way ANOVA with a Tukey's *post hoc* test).

TCAP-1 acts on the MEK-ERK1/2 pathway to induce polymerization of f-actin via activation of p90RSK and filamin A, leading to changes in neuronal cell cytoskeletal organization and morphology (22). If TCAP-1 is a ligand of LPHN1, then it is possible that its actions on this pathway occur through LPHN1 and its associated G proteins. To examine whether TCAP-1 induces cytoskeletal changes through an interaction with LPHN1, HEK-WT, and HEK-LPHN1-S cells were treated with either 100 nM mTCAP-1 or vehicle (control) for 60 min, and the cytoskeletal profile of the cells was observed (Figure 8). Vehicle-treated HEK-WT cells showed a strong f-actin signal at the cell perimeter as well as between adjacent cells (Figure 8C). This pattern was not seen in vehicle-treated HEK-LPHN1-S cells, which had a much weaker f-actin signal at the cell perimeter and little to no signal between adjacent cells (Figure 8M). mTCAP-1-treated HEK-WT cells showed little differences in f-actin labeling compared to vehicle-treated HEK-WT cells and had very few f-actin projections (Figure 8H). In contrast, mTCAP-1 treatment had a strong effect on f-actin expression in HEK-LPHN1-S cells (Figure 8R). These cells showed a much greater degree of f-actin labeling compared to those treated with vehicle, with a high amount of f-actin present throughout the cytosol of individual cells as well as between clustering cells. mTCAP-1-treated HEK-LPHN1-S cells also had more f-actin projections extending from them than vehicle-treated cells (Figure 8T, white arrows).

The expression of f-actin in these cells was again quantified as a function of the number of red pixels per cell, indicative of the degree of f-actin staining by Phalloidin (Figure 8U). No significant difference in red fluorescence was observed between HEK-WT cells treated with mTCAP-1 or vehicle, indicating no difference in their f-actin expression (WT + Veh: 986.706 ± 65.626 pixels/cell; WT + mTCAP-1: 823.586 ± 78.778 pixels/cell; *p* > 0.05). In contrast, mTCAP-1-treated HEK-LPHN1-S cells showed a 343% increase in red pixels per cell, indicating an increase in f-actin compared to vehicle-treated HEK-LPHN1-S cells (LPHN1-S + Veh: 175.314 ± 22.488 pixels/cell; LPHN1-S + mTCAP-1: 777.063 ± 49.511 pixels/cell; *p* < 0.0001). Furthermore, vehicle-treated HEK-LPHN1-S cells again showed a significantly decreased expression of f-actin compared to vehicle-treated HEK-WT cells, having ~82% less red pixels per cell than their wild-type counterpart (WT + Veh: 986.706 ± 65.626 pixels/cell; LPHN1-S + Veh: 175.314 ± 22.488 pixels/cell).

### Changes in Cell Morphology Upon Treatment With TCAP-1

Previous studies have indicated that LPHN1 and teneurin form an adhesion complex between adjacent cells, leading to increased formation of cell-cell contacts and aggregates (8). If their total volume is not affected, as cells cluster closer together due to an increase in cell-to-cell points of contact between LPHN1 and teneurin, changes in cell dimensions can be expected to occur, such as decreases in width and length and an increase in height. Similarly, if TCAP-1 is acting on the LPHN1-teneurin adhesion complex, a reversion to wild-type cell dimensions upon mTCAP-1 treatment may be expected. To determine if this is the case in HEK293 cells, the heights of HEK-WT and HEK-LPHN1-S cells upon treatment with 100 nM mTCAP-1 or vehicle for 60 min were measured (Figure 8V). For HEK-WT cells, no significant differences in cell height were observed between treatment with mTCAP-1 and vehicle (HEK-WT + Veh: 5.708 ± 0.180 μm; HEK-WT + mTCAP-1: 5.976 ± 0.180 μm). However, vehicle-treated HEK-LPHN1-S cells had a height of 8.113 ± 0.298 μm, which was significantly larger than that of HEK-WT cells treated with either vehicle or mTCAP-1 (*p* < 0.0001). Treatment of HEK-LPHN1-S cells with mTCAP-1 resulted in a 39% decrease in height to 4.968 ± 0.199 μm (*p* < 0.0001). There was no significant difference in height between vehicle-treated HEK-WT cells and mTCAP-1-treated HEK-LPHN1-S cells (HEK-WT + Veh: 5.708 ± 0.180 μm; HEK-LPHN1-S + mTCAP-1: 4.968 ± 0.199 μm; *p* > 0.05).

## Discussion

The data presented in this study provides novel evidence that the TCAP-1 region of teneurin-1 associates directly with LPHN1, and, as a diffusible peptide, can modulate cell-to-cell adhesion and cytoskeletal dynamics. Specifically, a GFP-tagged TCAP-1 construct co-immunoprecipitated with a portion of the LPHN1 extracellular domain containing the LPHN1 HBD, indicating affinity between the two. This is the first study to show that an Adhesion-type GPCR binding region has the potential to bind a ligand related to the Secretin family of peptides. When treated with FITC-K37-mTCAP-1, HEK-LPHN1-S cells had a higher level of co-localization of the peptide with LPHN1 than HEK-WT cells. Morphologically, over-expression of LPHN1 modulated cell size and decreased f-actin expression in HEK-LPHN1-S cells relative to the HEK-WT cells. mTCAP-1 treatment had little effect on the morphology of HEK-WT cells, whereas treatment of HEK-LPHN1-S cells resulted in changes to the cytoskeletal organization consistent with previous observations of TCAP-1 function. Together, these studies link the actions of synthetic TCAP-1 described in previous studies with the actions of the teneurin-LPHN complex as well as results reported in recent studies on the structure of teneurin and the LPHNs.

### TCAP-1 Interaction With LPHN1 at the Receptor Hormone Binding Domain

Since their discovery, the TCAP peptides were established to have major sequence identity initially with CRF and calcitonin, and subsequently, to a lesser but still compelling degree, with other members of the Secretin peptide family (16–18). The phylogenetic rationale for this relationship is not clear, currently. However, Secretin peptides are the ligands of Secretin GPCRs, and interact with their respective receptors at the receptor HBD. CRF itself, for example, binds to the HBD of its cognate receptors, CRFR1 and CRFR2, both of which belong to the Secretin family of GPCRs (35–38). Upon their discovery, the LPHNs were initially classified as members of the Secretin GPCR family due to their sequence similarity to the CRFR HBD and transmembrane region, but were later reclassified to the much more ancient Adhesion GPCR family (31, 35, 36, 39). The Adhesion GPCRs are themselves ancestral to the Secretin GPCRs, which suggests that the Secretin GPCRs inherited their ligand-binding HBDs from their Adhesion GPCR ancestors (36). Thus, given the similarity between the TCAP-1 and CRF peptides, and the structural similarities and phylogenetic histories of their respective receptors, we postulated that TCAP-1 interacts and with LPHN1 at its HBD.

To further characterize the similarities between the HBDs of LPHN and the Secretin family GPCRs, the LPHN1-3 HBD amino acid sequences were compared to those of the CRF receptors CRFR1 and CRFR2 and the calcitonin receptors CALCR and CGRPR (Figure 2A). These receptors were chosen as they are the most ancient of the Secretin GPCRs and the most closely related to the Adhesion GPCR family (36, 40). The comparison showed key sequence similarities at several HBD sites known to be critical for Secretin GPCR ligand binding (41–44). For example, in LPHN1, the cysteine residues C475, C499, and C511 are conserved in CRFR1 as C44, C68, and C87. Mutation of these residues in CRFR1 ablates CRF binding to the receptor (43). Studies using double-mutation of C68 and C87 in CRFR1 suggest that these residues form a disulfide bridge with each other, likely shaping the structure of the CRFR1 binding pocket (43). Moreover, the side chain of CRFR1 G64/CRFR2 G90 takes part in ligand interaction (42), and is conserved in all three LPHN paralogues. The strong conservation of these residues suggests their functional significance may also be conserved, potentially acting to form a LPHN1 HBD ligand-binding pocket. Interestingly, studies by Krasnoperov et al. (32) showed that multiple LPHN1 mutants lacking the HBD were unable to bind α-latrotoxin, further indicating the importance of this domain with respect to LPHN1 ligand interaction.

The potential ability of TCAP to interact with LPHN is consistent with previous studies, but provides a novel understanding of this relationship. The C-terminal region of teneurin-2, containing the TCAP-2 sequence, does bind to LPHN1 (27, 28); however, evidence of a direct interaction between LPHN and TCAP itself was not yet established in initial studies reporting LPHN1-teneurin-2 binding. Given these results and the conservation of the ligand and HBD structure described above, a co-IP assay was performed in which HEK293 cells were co-transfected with GFP-pro-mTCAP-1 or GFP-mTCAP-1, as well as constructs encompassing different portions of the LPHN1 HBD region (Figure 1). The TCAP-based constructs were designed according to the expected full-length TCAP-1 mRNA established from a previous study (19). The GFP-pro-mTCAP-1, based on the full-length TCAP-1 mRNA, was not detected in any eluates, suggesting that no interaction occurs between the TCAP-1 pro-peptide and LPHN1 HBD. In contrast, the GFP-mTCAP-1 construct, based on the putative 41-mer TCAP-1 region, was present as a band at approximately 30 kDa in the V444-E634 eluate and, to a lesser degree, in the V443-Q579 eluate (Figure 3, IPs, red arrow). This corresponds to the 30 kDa band observed for GFP-mTCAP-1 in the eluate inputs (Figure 3, inputs), and is consistent with a protein composed of the 25 kDa GFP and the 4.7 kDa mature TCAP-1 peptide. These results indicate that the mature TCAP-1 peptide may interact with LPHN1, most likely with a segment encompassing HBD residues V444 to E634. The presence of a weaker GFP-mTCAP-1 band in the V444-Q579 eluate suggests that this specific region of LPHN1 may be able to bind the mature TCAP-1 peptide at a lower affinity. Thus, although LPHN1 residues V444-to Q579 participate in ligand binding, they may represent only a partial binding pocket, whereas residues V444 to E634 may provide a more complete binding domain with which LPHN1 ligands can interact. It is important to note that both HBD constructs also contained a portion of the LPHN1 GAIN domain (Figure 1). This domain is unique to the Adhesion family of GPCRs and is thought to have a role in the transduction of conformational changes to the receptor transmembrane region, ultimately leading to induction of intracellular responses upon ligand-receptor binding (45). As the V444-E634 construct included a greater proportion of the GAIN domain than V444-Q597, it is likely that elements of the LPHN1 GAIN domain do play a role in peptide-binding. This may occur either through a direct contribution to the binding pocket, or by indirect stabilization of the tertiary HBD structure. Further studies will be required to ascertain the domains involved in the formation of the LPHN1 peptide-binding pocket.

### TCAP-1 Co-localizes With LPHN1 at the Cell Membrane

Having established that TCAP-1 can interact with the HBD/GAIN region of LPHN1, the next step was to determine if TCAP-1 could co-localize with LPHN1-overexpressing HEK293 cells. To confirm successful expression of the LPHN1-S vector, HEK-WT, and HEK-LPHN1-S cells were analyzed using western blot, which showed a strong band of about 120 kDa for HEK-LPHN1-S cells only (Figure 4A). This is consistent with a band at around 120 kDa found by Davletov et al. (29) in their study describing the initial discovery of LPHN1 as a receptor for α-latrotoxin. To corroborate this, and to observe the expression pattern of the LPHN1-S construct, an ICC analysis was performed using fluorescently-tagged antibodies targeting the FLAG tag at the construct N-terminal (Figure 4C). A strong signal was observed at the cell membrane in HEK-LPHN1-S cells, confirming successful transfection and high expression of the construct. No such signal was evident in the HEK-WT cells or the transfection control HEK-puro cells. Thus, HEK-WT and HEK-LPHN1-S cells were used to further investigate the binding of TCAP-1 and LPHN1, and the resultant downstream signaling effects.

HEK-WT and HEK-LPHN1-S cells were treated with FITC-K37-mTCAP-1 followed by a fluorescent antibody targeting the LPHN1-S construct, and the degree of TCAP-1 uptake and TCAP-1/LPHN1 co-localization in each cell type was observed (Figure 5). HEK-WT cells showed minimal uptake of FITC-K37-mTCAP-1, whereas HEK-LPHN1-S cells had a significant FITC-K37-mTCAP-1 signal at the plasma membrane. A marked overlap of FITC-K37-mTCAP-1 and LPHN1 at the cell membrane was also observed, indicating that the two are proximal to each other. As the endogenous presence of other LPHN isoforms was not assessed in the two cell lines used in this study, it is possible that TCAP-1 may be binding with these proteins as well. However, the significant difference in TCAP-1 uptake between HEK-WT and HEK-LPHN1-S cells and the strong overlap of the LPHN1-S and TCAP-1 fluorescence signals observed here indicate that TCAP-1 is most likely primarily interacting with LPHN1 in HEK-LPHN1-S cells. Despite these findings, our model renders an incomplete picture with respect to interaction between teneurin/TCAP and LPHN. Because there are four forms of teneurins/TCAPs and at least three LPHN paralogues in the vertebrate genome, it has been a challenge to find the perfect model to understand the interactions among the teneurins and LPHNs. We cannot discount the possibility that TCAP interacts with other LPHNs or indeed other receptor systems. However, it is important to note that this is the first study to report such an interaction between LPHN1 and the TCAP-1 region of teneurin-1. Recently, new studies of TCAP-1 in skeletal cells indicate that siRNA and CRISPR knockdowns of the LPHN1 receptor ablate TCAP-1 secondary messenger activity (D'Aquila et al., manuscript in preparation). Together, these studies indicate that TCAP-1 may interact with LPHNs.

It is important to note that particular regions of the cell membrane showed combined LPHN1 and TCAP-1 signals, whereas others showed only a LPHN1 signal or only a FITC-K37-mTCAP-1 signal (Figure 5II). This is the first study to show a potential interaction of the putative TCAP-1 peptide with LPHN1; however, our findings indicate that although TCAP-1 can interact with LPHN1, it may do so under only certain structural orientations. Our study differs from previous studies investigating the interaction between LPHN and teneurin (8, 27, 28) in that the soluble FITC-K37-mTCAP-1 peptide used here was introduced into an environment where intercellular interactions between LPHN1 and its binding partners, such as teneurin, may have already been established. It is possible that the TCAP-1 peptide possesses less affinity for the receptor to compete with existing teneurin-LPHN1 interactions and thus preferentially binds to LPHN1 receptors that are not occupied by the full-length teneurin proteins. If this is the case, regions with TCAP-1-LPHN1 interaction would be co-labeled with the fluorescent tags for both, whereas regions of teneurin-LPHN1 interaction would present only LPHN1 labeling (Figures 5II,C,H), accounting for many of the fluorescence patterns observed here. Similarly, regions of the cell membrane with only a FITC-K37-mTCAP-1 signal were observed (Figures 5II,B,G). As endogenous expression of the three LPHN isoforms in HEK-WT and HEK-LPHN1-S cells was not examined, it is possible that TCAP-1 may also be interacting with another LPHN isoform, or another protein at the cell membrane. It is not uncommon for peptides to bind multiple isoforms of their receptors; CRF is able to bind both CRFR1 and CRFR2 (37, 38), and α-latrotoxin binding has been observed for both LPHN1 and LPHN2 (29, 46). Interaction with other endogenously expressed LPHN isoforms would also account for the slight degree of FITC-K37-mTCAP-1 fluorescence observed in HEK-WT cells. In addition, a recent study by Li et al. (30) on the structure of the teneurin/TCAP region indicates that, *in vivo*, the TCAP region of the teneurins may be partially hidden by the teneurin protein. If so, such an arrangement may act to reduce the immunoreactive TCAP-1 signal.

### Morphological Effects of LPHN1 Expression and TCAP-1 Treatment in HEK293 Cells

To assess the effects of LPHN1 over-expression in HEK293 cells and the effects of TCAP-1 treatment on the HEK-WT and HEK-LPHN1-S cell lines, several components of cell morphology were examined. Initial observations indicated that HEK-LPHN1-S were significantly smaller compared to HEK-WT cells, as shown by measurements of nuclear and whole cell area and perimeter (Figures 6I–L). However, these changes were quantified on a two-dimensional basis and further investigation showed that vehicle-treated HEK-LPHN1-S cells are taller than vehicle-treated HEK-WT cells. This suggests that the observed changes in cell size are likely to be purely morphological, with total cell volume being unaffected.

To date, a role in pathways that influence mammalian cell size, such as those associated with mTOR and P13K (47), has not been reported for LPHN1; however adhesion roles have been individually established for both LPHN1 and its binding partners, and the formation of the teneurin-LPHN trans-synaptic complex can increase adhesion between cells (8, 13, 48). Increased expression of LPHN1 could lead to increased formation of intercellular adhesion complexes, leading to cells clustering together more tightly. Within a confined space, this would result in a shift from a spherical or cubic cell shape to one that is more columnar, suggesting that the morphological differences observed between HEK-WT and HEK-LPHN1-S cells are simply due to increased adhesion between HEK-LPHN1-S cells. These cells also had a significantly reduced expression of f-actin and had fewer f-actin projections compared to HEK-WT cells (Figure 7). As HEK-LPHN1-S cells cluster closer together, a reduction in cytoskeletal elements between cells is to be expected. To date, a role for LPHN in cytoskeletal modulation has not been established; however, knock-down of LPHN2 in chicken cardiac tissue results in differential expression of 37 cytoskeletal genes (49). Together, these data suggest a potential role for LPHN1 in the regulation of f-actin polymerization.

As TCAP-1 induces cytoskeletal changes in neurons by modulating f-actin polymerization (19–21), the next step was to investigate the effects of TCAP-1 treatment on the morphology and cytoskeletal profiles of HEK-WT and HEK-LPHN1-S cells. Treatment with mTCAP-1 had no significant effects on HEK-WT actin polymerization, whereas it induced a significant f-actin increase in HEK-LPHN1-S cells. HEK-LPHN1-S cells also had more f-actin projections than their wild-type counterparts. This is consistent with the actions of TCAP-1 on the cytoskeletal arrangement of neuronal cells. TCAP-1-treated murine hypothalamic neurons show elongated neurites and increased expression of cytoskeletal components (20), whereas primary hippocampal neurons have greater neurite number, larger axon bundles, and changes in their dendritic arborization (21). These cytoskeletal changes are due to ERK1/2-induced polymerization of f-actin and re-organization of microtubules (19). TCAP-1-activated ERK1/2 phosphorylates p90RSK, which can in turn phosphorylate filamin A, causing it to induce cross-linking and stabilize actin filaments in neuronal cells. The marked increase in the f-actin expression of the HEK-LPHN1-S cells but not of the HEK-WT cells suggests that this action of TCAP-1 on the cytoskeleton may occur via LPHN1. It is possible that over-expression of the LPHN1-S isoform affects the health and functioning of HEK293 cells in such a way that impacts their cytoskeletal components and cellular morphology and that treatment with TCAP-1 simply acts to sequester LPHN1 and thus reduce those effects. However, similar studies to this one regarding LPHN1-teneurin binding in HEK293 cells have previously been conducted with no evidence of such an effect (8), and cytoskeletal modulation is a well-documented effect of TCAP-1 (19–21), making this an unlikely interpretation of the data presented here. Thus, taken together, these results are the first to show that TCAP-1 induces its effects through an interaction with LPHN1, indicating that TCAP-1 and LPHN1 form an endogenous ligand-receptor pair. This is particularly important, as it is also the first time that a role in cytoskeletal modulation has been reported for LPHN1.

### TCAP and LPHN as an Evolutionarily Ancient Receptor-Ligand Pair

Teneurin/TCAP and LPHN comprise the only known trans-synaptic pair to be conserved in both vertebrates and invertebrates (50), and they appear to have a shared evolutionary history. TCAP is an ancient peptide related to CRF and other members of the Secretin peptide family (18, 51, 52), whereas LPHN belongs to the Adhesion GPCRs, from which the Secretin GPCRs evolved (35, 36). Furthermore, both are found in the choanoflagellate, a single-celled ancestor of the metazoans (30, 53–55), where TCAP is hypothesized to have been acquired from a prokaryote genome via a horizontal gene transfer of an ancestral teneurin-like gene (56). Interestingly, the teneurins are structurally similar to bacterial polymorphic proteinaceous toxins, which possess a soluble toxin payload at their C-terminus that can be released into target cells (30, 53, 56). This payload is highly conserved and corresponds to the TCAP portion of teneurin, further indicating the similarities between these proteins and highlighting the extended evolutionary history of the teneurins.

## Conclusion and Final Remarks

In summary, TCAP-1 is a highly bioactive peptide with actions both *in vitro* and *in vivo*. *In vitro*, it is associated with multiple signal transduction systems, such as the MEK-ERK1/2 pathway, and can modulate the cytoskeleton (20–22). *In vivo*, TCAP-1 affects anxiety- and stress-related behaviors in a manner that is dependent, in part, on the baseline emotionality of animals, with different responses to treatment being observed between high baseline and low baseline animals (17). Previous studies suggest that the teneurins and LPHN represent a conserved trans-synaptic ligand-receptor pair with a number of intercellular actions (8, 27, 28). This study indicates that the TCAP region of teneurin, as a soluble peptide, also plays a role in this interaction and that its roles in cytoskeletal remodeling occur in part via LPHN1. This sets the stage for future research to further elucidate the actions of TCAP-1 at both cellular and behavioral levels.

## Author Contributions

MH contributed to experimental design, collection, analysis, and interpretation of the data, writing of the manuscript and subsequent revisions of the manuscript. DB-L contributed to experimental design, performed cell transfections, and collected and aided in the analysis and interpretation of the data. DL supervised the project, contributed to experimental design, interpretation of the data, and writing and revision of the manuscript. All authors have reviewed and approved of the final draft of the manuscript.

### Conflict of Interest Statement

DL is a co-founder and chief scientific advisor of Protagenic Therapeutics Inc. The remaining authors declare that the research was conducted in the absence of any commercial or financial relationships that could be construed as a potential conflict of interest.
